# Evaluation of the correlation between frailty and sleep quality among elderly patients with osteoporosis: a cross-sectional study

**DOI:** 10.1186/s12877-022-03285-z

**Published:** 2022-07-19

**Authors:** Xiaoru Xu, Xiaoping Zhou, Wenjing Liu, Qing Ma, Xuexue Deng, Ronghua Fang

**Affiliations:** West China School of Nursing/Department of International Medical Center, West China Hospital, Sichuan University, Chengdu, China

**Keywords:** Osteoporosis, Elderly, Frailty, Sleep quality, Correlation

## Abstract

**Background:**

The incidence of osteoporosis increases with age. Frailty is a distinct characteristic seen in older osteoporosis patients. Poor sleep quality is common in elderly individuals. However, there are few studies on the correlation between frailty and sleep quality in elderly patients with osteoporosis in China.

**Methods:**

This cross-sectional study was conducted from December 8, 2020, to April 30, 2021. A total of 247 patients who met the inclusion and exclusion criteria were recruited in outpatient departments at West China Hospital. A self-designed demographic data questionnaire, the Pittsburgh Sleep Quality Index (PSQI), and the Frailty Phenotype scale were used to evaluate the sleep quality and frailty of the participants. Multivariate logistic regression was performed to evaluate the factors affecting frailty and sleep quality in osteoporosis patients.

**Results:**

A total of 231 valid questionnaires were statistically analysed. The mean frailty score was 3.2 ± 1.6 and a total of 152 (65.8%) were frailty. The mean PSQI score was 11.9 ± 4.5 and a total of 183 (79.2%) patients had poor sleep quality (PSQI > 7). Multiple logistic regression showed that female, pain, polypharmacy, activities of daily living status (ADLs), and sleep quality were independent influencing factors for frailty, while comorbidity, ADLs and frailty status were independent influencing factors for sleep quality.

**Conclusion:**

We found that frailty and sleep quality were prevalent and that frailty was positively correlated with the PSQI score in elderly patients with osteoporosis in China. The higher the frailty score is, the worse the sleep quality. It means the frailer old patients were, the worse their sleep quality, and poor sleep quality may increase the risk of frailty in elderly patients with osteoporosis. To manage elderly patients with osteoporosis effectively, medical staff should pay attention to frailty, sleep quality and its influencing factors.

## Background

Osteoporosis (OP) is a systemic bone disease characterized by reduced bone mass and damage to the bone microstructure, resulting in increased bone fragility and fracture, and the incidence of the disease increases with age [1]. OP causes acute and chronic pain, height changes, and reduced activities of daily living (ADL), and increases the risk of hospitalization, individual socioeconomic burden, morbidity, and mortality [1, 2]. There were 20 million cases of osteoporosis in EU6 countries (France, Germany, Italy, Spain, the United Kingdom and Sweden) in 2015, 15.8 million of these cases involved women, and 4.2 million involved men [1]. The prevalence of OP in people > 60 years of age was 36%, comprising 23% males and 49% females in China [3]. The number of patients with OP-related fractures in China will reach 5.99 million, and medical expenditures will reach $25.43 billion by 2050 [4]. A previous study showed that OP was associated with an increased risk of death for 5–10 years after the onset of a fragility fracture in people > 60 years of age [5].

Frailty is caused by the cumulative decline of multiple physiological systems associated with age and is a multidimensional syndrome, including physical, social and cognitive dimensions [6]. The prevalence of frailty ranges from 4.0–59.1% globally and 7.0–28.0% in China, and the trend increases each year [6, 7]. Frail elderly individuals are more likely to suffer from falls, disability, delirium, cognitive impairment, hospitalization, and even death [8].

The overall prevalence rates of poor sleep quality ≥ 60 years were 41.5–49.7% in China [9, 10] and 17–31% in 16 European countries [11]. Poor sleep quality and sleep disorder can increase the risk of obesity, cardiovascular disease, type 2 diabetes mellitus, and depression [12].

The literature showed a bidirectional relationship between frailty and sleep quality in elderly persons. Frailty is often accompanied by a drop in growth hormone levels, leading to a decline in muscle mass and strength [13], and a drop in growth hormone levels may also be associated with poor sleep quality [14]. In addition, poor sleep quality can cause internal changes in the body [15], and decreases hormone levels, inflammatory molecules, and renal insufficiency can lead to protein degradation and muscle proteolysis, thus increases the risk of frailty in older adults [15–17]. That is, poor sleep quality affects endocrine and metabolic functions, and increases the risk of frailty [18, 19]. Conversely, frailty may decrease sleep quality [20].

Frailty and poor sleep quality are important characteristics in elderly patients with OP [21, 22]. Previous studies showed that the prevalence of frailty and poor sleep quality in the elderly adults with OP was 74.77% and 50%, respectively [23, 24], which was higher than that in common elderly persons [6, 7, 9, 10] in China. In recent years, an increasing number of studies have been conducted on sleep quality and OP, or frailty and OP for older adults, However, there are few studies on sleep quality and frailty, and the relationship between frailty and sleep quality in elderly patients with OP in China.

Thus, the present study evaluated the association between frailty and sleep quality among elderly patients with OP in China.

## Methods

### Population and data

A questionnaire survey was conducted among elderly patients with OP who visited the West China Hospital of Sichuan University between December 8, 2020, and April 30, 2021. A cross-sectional study was conducted, and a total of 247 elderly outpatients with OP were invited to participate in this survey. The Ethics Committee of the West China Hospital of Sichuan University approved this research. Informed consent was obtained from all participants. Survey data were anonymously coded to ensure that identifying information remained confidential.

All study participants met the diagnostic criteria of the guidelines for the diagnosis and treatment of OP in elderly individuals in China (2018 Edition) [25]. Bone mineral density (BMD) at the lumbar spine (L1-L4) and the femoral neck or 1/3 of the distal radius was measured for each participant. A T-score of any bone site ≥ -1.0 is considered normal; -2.5 < T-score < -1.0 is considered osteopenia; T-score ≤ -2.5 is considered OP [25]. The inclusion criteria were as follows: diagnosis of OP (bone density examination through dual-energy X-ray absorptiometry, DXA); age ≥ 60 years; intact cognitive ability; agreement to participate in this survey; no poor sleep quality due to alcohol use, pharmacotherapy or disease; and no family history of sleep problems. The exclusion criteria were as follows: parathyroid and adrenal gland corticosteroids, malignant tumor, and thyroid.

There were three types of questionnaires included: demographic data, the Frailty Phenotype scale and the Pittsburgh Sleep Quality Index (PSQI). For data collection, researchers conducted face-to-face interviews with each participant in the outpatient department of the West China Hospital, Sichuan University. Before the survey, investigators received uniform training on the correct methods of data collection and pass the test. The investigator used uniform language to explain the purpose, significance and relevant instructions of the investigation and obtained a signed consent form prior to conducting the investigation. The researchers gave each participant a paper questionnaire, which was completed independently and collected immediately.

The database was built using EpiData 3.1 software (EpiData–Comprehensive Data Management and Basic Statistical Analysis System, EpiData Association, Odense, Denmark) and then double-checked. Incomplete questionnaires were deleted.

### Outcomes

Frailty was assessed by using Fried's frailty phenotype scale, which includes five elements: unintentional weight loss, self-reported exhaustion, slow walking speed, weakness, and low physical activity [26]. Unintentional weight loss was defined as unintentional weight loss ≥ 4.5 kg or body weight ≥ 5% within 1 year (at follow-up, direct weight measurement). Self-reported exhaustion is used to determine how often the patient's condition has happened by asking two questions (“I cannot walk” and “I feel like everything I do requires effort”) in the past week. Scores range from 0 to 3 based on frequency. 0 = little or none of time (< 1 day), 1 = Sometimes (1–2 days), 2 = often (3–4 days), 3 = most of time (> 4 days). If the score for any question is ≥ 2, it shows exhaustion. Slow walking speed is based on time to walk 4.57 m, adjusting for sex and standing height. The following conditions indicate slow walking speed: height (cm) and walking time (s) are, respectively, male, ≤ 173 cm and ≥ 7 s, > 173 cm and ≥ 6 s, and female ≤ 159 cm and ≥ 7 s, > 159 cm and ≥ 6 s. Weakness is measured by grip strength, using maximum handgrip strength of either hand (twice test for each; keep a standing position to measured), adjusted for gender and body mass index (BMI). The following conditions indicate weakness: BMI (kg/cm^2^) and grip strength (kg) are, respectively, ≤ 24.0 kg/cm^2^ and ≤ 29 kg, 24.1–26.0 kg/cm^2^ and ≤ 30 kg, 26.1–28.0 kg/cm^2^ and ≤ 30 kg, > 28.0 kg/cm^2^ and ≤ 32 kg, and female ≤ 23.0 kg/cm^2^ and ≤ 17 kg, 23.1–26.0 kg/cm^2^ and ≤ 17.3 kg, 26.1–29.0 kg/cm^2^ and ≤ 18 kg, > 29.0 kg/cm^2^ and ≤ 21 kg. Conditions of kilocalories (kals) expended per week indicate low physical activity: males < 383 kcal and females < 270 kcal. These five factors were scored on a scale from 0 to 1 (0 = no, 1 = yes). The total score was the sum of the scores for each element: 0 = nonfrail, 1–2 = prefrail, and ≥ 3 = frail [26].

The Chinese version of the PSQI, which was translated by Liu et al.,was used for assessing subjective sleep quality over the previous month, and the Cronbach’s α was 0.719 [27]. The PSQI includes 18 self-evaluation items, which comprise 7 aspects: sleep quality, sleep latency, sleep duration, sleep efficiency, sleep problems, use of sleep drugs and daytime dysfunction. Each component was scored on a Likert-type 4-point scale (0–3). The total score of the PSQI was summed to obtain a global score ranging from 0–21. PSQI ≤ 7 indicated normal sleep quality, and PSQI > 7 indicated poor sleep quality [28].

### Covariates

The socioeconomic and demographic factors selected were gender (male, female); age group (60–74 years, 75–89 years, and 90 years and older); marital status (married, unmarried or widowed); education status (primary school and below, junior school, high school, college and above); residence (alone/nursing home, cohabitant spouse, cohabitant with children); past occupation (manual labor, mental labor); and income (pension/social security, child support, other). We focused on those aged ≥ 60 years because the age of 60 is generally considered a sign of old age in China.

Health situation factors were smoking history (yes/no); drinking history (yes/no); history of fracture (yes/no); pain (yes/no); comorbidity means ≥ 2 conditions (yes/no); polypharmacy considers taking ≥ 5 medications simultaneously (yes/no); participation in social activity (no, 1 to 2 times/year, 1 to 2 times/month, 1 to 2 times/week); and BMI, which was calculated as weight (kg) divided by the square of height (m). We categorized BMI into three groups: low weight, BMI ≤ 18.5 kg/m^2^; normal weight, 18.5 kg/m^2^ < BMI < 23.9 kg/m^2^; and overweight/obesity, BMI ≥ 24 kg/m^2^ [20]. We used the modified Barthel index (MBI) to assess older adults’ ADLs, which includes feeding, bathing, grooming, dressing, bowels and bladder control, toilet use, transfer, mobility, and climbing stairs. A total score ranges from 0–100, with higher scores indicating better ADL. We divided ADL into four statuses: no impairment = 100; mild impairment = 61–99; moderate impairment = 41–60; and severe impairment = 0–40 [29].

### Statistical analysis

SPSS software (Version 20.0 IBM Inc., Armonk, NY) was used for the statistical analyses. Continuous data are presented as the means and standard deviations, and categorical data are presented as frequencies and percentages. χ^2^ tests were used to compare groups. Logistic regression was used to analyse the influencing factors of frailty and sleep quality. Nonfrail or frail (0–2 was nonfrail and ≥ 3 was frail) was the dependent variable, and considering the interference factors, the univariate analysis of demographic data, gender, age, BMI, marital status, education, residence, past occupation, income, smoking history, drinking history, history of fracture, pain, comorbidity, polypharmacy, participation in social activity, ADLs and sleep quality with *P* < 0.2 were independent variables. Normal sleep quality or poor sleep quality was the dependent variable, and the inclusion method of independent variables was the same as that of frailty influencing factor analysis. The correlation between frailty and PSQI scores was analyzed. Pearson correlation analysis was used if the data followed a normal distribution, and Spearman correlation analysis was used if the data did not follow a normal distribution. A *p* value < 0.05 was considered statistically significant.

## Results

### Participants

**Fig. 1:**
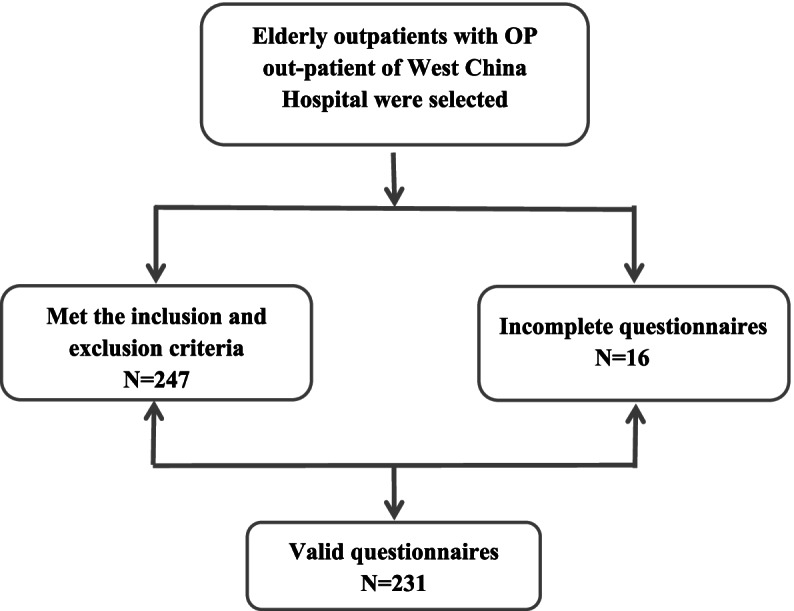
The step flow diagram including all steps from screening to study

A total of 247 elderly individuals met the inclusion and exclusion criteria, the number of complete questionnaires was 231 (Fig. 1). The valid rate was 93.5%. The sample had the following characteristics: the majority of participants were female and between 60–74 years old; married, 79.7%; junior school, 25.1%; cohabitant with children, 54.1%; manual labor, 59.7%; history of fracture, 33.3%; pain, 90.9%; comorbidity, 61.9%; polypharmacy, 20.8%; no participation in social activity, 43.3%; and moderate impairment ADLs, 8.7% (Table 1).

**Table 1:** Characteristics of the participants based on different frailty (*n* = 231)

Variables	n(%)	Nonfrail n(%)	Prefrail n(%)	Frail n(%)	X^2^	*P*
Gender					23.379	0.000
Male	87 (37.7)	6 (6.9)	39 (44.8)	42 (48.3)		
Female	144 (62.3)	11 (7.6)	23 (16.0)	110 (76.4)		
Age, y					25.805	0.000
60–74	152 (65.8)	17 (11.2)	51 (33.6)	84 (55.3)		
75–89	70 (30.3)	0 (0.0)	8 (11.4)	62 (88.6)		
≥ 90	9 (3.9)	0 (0.0)	3 (33.3)	6 (66.7)		
BMI(kg/m^2^)					15.412	0.004
Low weight	24 (10.4)	0 (0.0)	1 (4.2)	23 (95.8)		
Normal weight	127 (55.0)	10 (7.9)	31 (24.4)	86 (67.7)		
Overweight/obesity	80 (34.6)	7 (8.7)	30 (37.5)	43 (53.8)		
Marital status					2.384	0.304
Married	184 (79.7)	16 (8.7)	49 (26.6)	119 (64.7)		
Unmarried or widowed	47 (20.4)	1 (2.1)	13 (27.7)	33 (70.2)		
Education					22.151	0.001
Primary school and below	77 (33.3)	7 (9.1)	20 (26.0)	50 (64.9)		
Junior school	58 (25.1)	3 (5.2)	8 (13.8)	47 (81.0)		
High school	49 (21.2)	0 (0.0)	14 (28.6)	35 (71.4)		
College and above	47 (20.4)	7 (14.9)	20 (42.6)	20 (42.6)		
Residence					17.074	0.002
Alone/nursing home	15 (6.5)	1 (6.7)	6 (40.0)	8 (53.3)		
Cohabitant spouse	91 (39.4)	12 (13.2)	31 (34.1)	48 (52.7)		
Cohabitate with children	125 (54.1)	4 (3.2)	25 (20.0)	96 (76.8)		
Past occupation					0.086	0.958
Manual labor	138 (59.7)	10 (7.2)	38 (27.5)	90 (65.3)		
Mental labor	93 (40.3)	7 (7.5)	24 (25.8)	62(66.7)		
Income					5.197	0.268
Pension/Social security	148 (64.1)	13 (8.8)	38 (25.7)	97 (65.5)		
Child support	71 (30.7)	3 (4.2)	18 (25.4)	50 (70.4)		
Other	12 (5.2)	1 (8.3)	6 (50.0)	5 (41.7)		
Smoking history					4.146	0.126
No	191 (82.7)	11 (5.8)	52 (27.2)	128 (67.0)		
Yes	40 (17.3)	6 (15.0)	10 (25.0)	24 (60.0)		
Drinking history					10.140	0.006
No	191 (82.7)	11 (5.8)	46 (24.1)	134 (70.2)		
Yes	40 (17.3)	6 (15.0)	16 (40.0)	18 (45.0)		
History of fracture					9.946	0.007
No	154 (66.7)	15 (9.7)	48 (31.2)	91 (59.1)		
Yes	77 (33.3)	2 (2.6)	14 (18.2)	61 (79.2)		
Pain					24.071	0.000
No	21 (9.1)	5 (23.8)	12 (57.1)	4 (19.0)		
Yes	210 (90.9)	12 (5.7)	50 (23.8)	148 (70.5)		
Comorbidity					3.814	0.149
No	88 (38.1)	6 (6.8)	30 (34.1)	52 (59.1)		
Yes	143 (61.9)	11 (7.7)	32 (22.4)	100 (69.9)		
Polypharmacy					18.023	0.000
No	183 (79.2)	16 (8.7)	59 (32.2)	108(59.0)		
Yes	48 (20.8)	1 (2.1)	3 (6.2)	44 (91.7)		
Participate in social activity					9.787	0.134
No	100 (43.3)	4 (4.0)	21 (21.0)	75 (75.0)		
1 to 2 times/year	108 (46.8)	11 (10.2)	31 (28.7)	66 (61.1)		
1 to 2 times/month	16 (6.9)	1 (6.2)	7 (43.8)	8 (50.0)		
1 to 2 times/week	7 (3.0)	1 (14.3)	3 (42.9)	3 (42.9)		
ADLs					99.243	0.000
No impairment	83 (35.9)	16 (19.3)	46 (55.4)	21 (25.3)		
Mild impairment	117 (50.7)	1 (0.9)	16 (13.7)	100 (85.5)		
Moderate impairment	20 (8.7)	0 (0.0)	0 (0.0)	20 (100.0)		
Severe impairment	11 (4.8)	0 (0.0)	0 (0.0)	11 (100.0)		
Sleep quality					32.510	0.000
Nomal sleep quality ^A^	48 (20.8)	8 (16.7)	25 (52.1)	15 (31.2)		
Poor sleep quality ^B^	183 (79.2)	9 (4.9)	37 (20.2)	137 (74.9)		

A: Normal sleep quality indicates PSQI ≤ 7; B: poor sleep quality indicates PSQI > 7

### Demographic characteristics of the participants and frailty

The characteristics of the participants based on different degrees of frailty are shown in Table 1. A total of 152 (65.8%) elderly patients with OP were frail (frailty scores ≥ 3), and the mean frailty score of the 231 elderly patients with OP was 3.2 ± 1.6. There were statistically significant differences between different degrees of frailty in elderly patients with OP across the following variables: gender, age, BMI, education, residence, drinking, history of fracture, pain, polypharmacy, ADLs, and sleep quality (*P* < 0.05). The following factors were associated with higher prevalence of frailty among participants: female, age 75–89 years, low weight, junior school, cohabitant with children, no drinking, history of fracture, pain, polypharmacy, moderate and severe impairment ADLs, and poor sleep quality.

### Demographic characteristics of the participants and PSQI

The characteristics of the participants based on differences in sleep quality are shown in Table 2. A total of 183 (79.2%) elderly patients with OP had poor sleep quality (PSQI > 7), and the mean PSQI score of the 231 participants was 11.9 ± 4.5. There were statistically significant differences among sleep quality in elderly patients with OP across the following variables: age, BMI, past occupation, pain, comorbidity, polypharmacy, participation in social activity, ADLs, and frailty phenotype (*P* < 0.05). The following factors were associated with higher prevalence of poor sleep quality: 75–89 years, low weight, mental labor, pain, comorbidity, polypharmacy, no participation in social activity, severe impairment of ADLs and frailty.

**Table 2:** Characteristics of the participants based on different sleep quality (*n* = 231)

Variables	Nomal sleep quality ^A^ n(%)	Poor sleep quanlity ^B^ n(%)	X^2^	*P*
Gender			0.956	0.403
Male	21 (24.1)	66 (75.9)		
Female	27 (18.8)	117 (81.2)		
Age, y			14.056	0.001
60–74	41 (27.0)	111 (73.0)		
75–89	4 (5.7)	66 (94.3)		
≥ 90	3 (33.3)	6 (66.7)		
BMI(kg/m^2^)			17.794	0.000
Low weight	3 (12.5)	21 (87.5)		
Normal weight	16 (12.6)	111 (87.4)		
Overweight/obesity	29 (36.2)	51 (63.8)		
Marital status			0.810	0.421
Married	36 (19.6)	148 (80.4)		
Unmarried or widowed	12 (25.5)	35 (74.5)		
Education			6.803	0.078
Primary school and below	22 (28.6)	55 (71.4)		
Junior school	8 (13.8)	50 (86.2)		
High school	12 (24.5)	37 (75.5)		
College and above	6 (12.8)	41 (87.2)		
Residence			4.438	0.109
Alone/nursing home	6 (40.0)	9 (60.0)		
Cohabitant spouse	15 (16.5)	76 (83.5)		
Cohabitate with children	27 (21.6)	98 (78.4)		
Past occupation			9.507	0.002
Manual labor	38 (27.5)	100 (72.5)		
Mental labor	10 (10.8)	83 (89.2)		
Income			1.280	0.527
Pension/Social security	29 (19.6)	119 (80.4)		
Child support	15 (21.1)	56 (78.9)		
Other	4 (33.3)	8 (66.7)		
Smoking history			0.982	0.395
No	6 (15.0)	34 (85.0)		
Yes	42 (22.0)	149 (78.0)		
Drinking history			0.982	0.395
No	6 (15.0)	34 (85.0)		
Yes	42 (22.0)	149 (78.0)		
History of fracture			0.473	0.606
No	34 (22.1)	120 (77.9)		
Yes	14 (18.2)	63 (81.8)		
Pain			14.014	0.001
No	11 (52.4)	10 (47.6)		
Yes	37 (17.6)	173 (82.4)		
Comorbidity			20.974	0.000
No	32(36.4)	56(63.6)		
Yes	16(11.2)	127(88.8)		
Polypharmacy			5.701	0.017
No	44 (24.0)	139 (76.0)		
Yes	4 (8.3)	44 (91.7)		
Participate in social activity			8.904	0.044
No	16 (16.0)	84 (84.0)		
1 to 2 times/year	23 (21.3)	85 (78.7)		
1 to 2 times/month	5 (31.2)	11 (68.8)		
1 to 2 times/week	4 (57.1)	3 (42.9)		
ADLs			29.594	0.000
No impairment	33 (39.8)	50 (60.2)		
Mild impairment	14 (12.0)	103 (88.0)		
Moderate impairment	1 (5.0)	19 (95.0)		
Severe impairment	0 (0.0)	11 (100.0)		
Frailty status			32.510	0.000
Nonfrail	8 (47.0)	9 (53.0)		
Prefrail	25 (40.3)	37 (59.7)		
Frail	15 (9.9)	137 (90.1)		

A: Normal sleep quality indicates PSQI ≤ 7; B: Poor sleep quality indicates PSQI > 7

### Logistic regression analysis of factors related to frailty

The influencing factors of frailty were analysed. Different degrees of frailty were the dependent variable, and variables with *P* < 0.2 in demographic data included gender, age, BMI, education, residence, smoking history, drinking history, history of fracture, pain, comorbidity, polypharmacy, participation in social activity, ADLs and sleep quality. Multiple logistic regression analysis showed that female, pain, polypharmacy, ADLs and sleep quality were the main influential factors related to frailty in elderly patients with OP (Table 3).

**Table 3:** Logistic regression analysis of factors related to frailty

Variables	b	Sb	Wald	*P*	OR(95% CI)
Female	1.541	0.431	12.803	0.000	4.67 (2.01, 10.86)
With pain	1.494	0.758	3.880	0.049	4.46 (1.01, 19.70)
Polypharmacy	1.137	0.458	6.155	0.013	3.12 (1.27, 7.65)
ADLs	-2.720	0.413	43.320	0.000	0.07 (0.03, 0.15)
Poor sleep quality	0.987	0.483	4.178	0.041	2.68 (1.04, 6.91)
Constant	0.507	2.252	0.051	0.822	

Poor sleep quality indicates PSQI > 7

Logistic regression analysis of factors related to sleep quality.

The influencing factors of sleep quality were analysed. Poor sleep quality and normal quality was the dependent variable, and variables with *P* < 0.2 in demographic data included age, BMI, education, residence, past occupation, pain, comorbidity, polypharmacy, participate in social activity, ADLs and frailty status. Multiple logistic regression analysis showed that comorbidity, ADLs and frailty status were the main influencing factors related to sleep quality for elderly patients with OP (Table 4).

**Table 4:** Logistic regression analysis of factors related to sleep quality

Variables	b	Sb	Wald	*P*	OR (95%CI)
Comorbidity	1.714	0.389	19.457	0.000	5.55 (2.59,11.90)
ADLs	-1.118	0.375	8.874	0.003	0.33 (0.16,0.68)
Frailty	0.761	0.318	5.727	0.017	2.14 (1.15,3.99)
Constant	0.684	1.879	0.133	0.716	

### Analysis of the correlation between frailty and sleep quality

The frailty and PSQI scores followed a normal distribution, and Pearson’s test was used to analyze the correlation between frailty and sleep quality. The results of the correlation analysis showed that frailty was positively correlated with sleep quality (*R* = 0.541, *P* = 0.000). The higher the frailty score is, the worse the sleep quality.

## Discussion

The present study showed that frailty was prevalent among elderly outpatients with OP in China. This prevalence was higher than that in community-dwelling older adults [30, 31]. Our research found that frailty occurred in 48.3% of male patients and 76.4% of female patients with OP, and the result was higher than those of other study findings [30, 32]. Aging leads to a decrease in the ability to initiate and maintain sleep, including more fragmented sleep, less total sleep time, and less slow wave sleep [33], which can explain why people between the ages of 75–89 have more poor sleep.

Our study showed that female, pain, polypharmacy, ADLs and poor sleep quality were the main influencing factors related to frailty in elderly patients with OP (Table 3). Females were 4.67 times more likely to be at risk of frailty than males. Females are prone to vitamin D deficiency due to the decrease in estrogen after menopause, which affects neuromuscular balance and muscle strength and is more likely to result in frailty [7, 34]. Patients with pain were 4.46 times more likely to have a risk of frailty than those without pain. Pain often brings adverse consequences to elderly individuals, including falls, anxiety, depression, sleep disturbances, isolation and sarcopenia [35]. Compared with older adults without pain, the more severe pain experienced by older adults, the greater is their decline in physical activity and autonomy, which increases the risk of individual sarcopenia and frailty [36]. Increasing evidence indicates that pain-related health outcomes are related to the onset and progression of frailty [35, 37]. Polypharmacy was 3.12 times more likely to increase the risk of frailty than no polypharmacy. The relationship among frailty, comorbidity and polypharmacy may go both ways [38]. The reason is that frailty is associated with certain chronic diseases and multimorbidity, which can lead to polypharmacy. At the same time, some drugs can cause weight loss, imbalance, functional degeneration and other undesirable outcomes and lead to poor outcomes [37–39]. We found that elderly individuals with moderate and severe impairment ADLs are more prone to frailty. Impaired ADLs can reduce health-promoting behaviors, and exercise motivation declines and forms a vicious cycle in elderly individuals, which increases the probability of frailty and results in overall diminished health [20, 40]. Poor sleep quality was 2.68 times more likely to be associated with the risk of frailty than normal sleep quality. Other studies have shown that poor sleep quality is involved in the frailty state in elderly individuals, which predicts frailty [20, 41].

We also found that comorbidities, ADLs and frailty were the main risk factors related to sleep quality for elderly patients with OP (Table 4). Other studies showed that comorbidity was a risk factor for sleep disorders and frailty [19, 25]. With the increase in comorbidity, polypharmacy may aggravate poor sleep in the elderly due to the cascade effect [42]. Our study showed that participants with impaired ADLs were prone to poor sleep quality, and the result is similar to that of Çavuşoğlu [18]. Impaired ADLs not only reduce quality of life but also cause certain psychological impacts on older people; in addition, social circles become narrow, and the absence of outside support generates poor sleep [18, 41].

Previous study have suggested that the frailer the old patients were, the more their sleep quality was affected [18]. Frailty may lead to sleep rhythm disorder, fatigue, reduced activity and other adverse living conditions, increasing sleepiness and thus aggravating sleep quality [18, 43]. Poor sleep quality can increase the risk of frailty in older adults [12]. Therefore, frailty may interact with sleep quality [19]. Our research also shows that frailty and sleep interact. Other studies have not reported a significant cross-sectional relationship between frailty and OP, but measuring frailty as a predictor of osteoporotic fractures in the elderly has been validated [8, 44, 45]. In other words, frailty can increase the risk of fracture in older patients with OP. Poor sleep quality may increase or decrease bone formation, bone mineral density (BMD), and osteoclast activities, consequently aggravating bone loss of the old patients with OP [46]. Therefore, the interaction between frailty and sleep quality may influence OP, which needs further study.

### Limitations

There are several limitations to our study that could affect our results. First, the convenience of the sample, modified by not having a randomized sampling method, resulted in a small sample size, considering that we are referring to Chinese elderly individuals with OP. Second, sleep quality measurement was assessed just by self-reported questions. Lack of objective sleep measurement and other sleep problem assessment (e.g. snoring, obstructive sleep apnea) are limitations. Another limitation relates to the recruitment of participants, in which the proportion of female patients was higher than that of male patients (62.3% vs. 37.7%). Because our subjects were elderly patients with OP, the results of frailty and sleep quality may be different from those of ordinary elderly individuals, and further research is still needed.

In fact, as a cross-sectional study, although the elderly were retired and lived in the same province, they still had different conditions: some cohabitated with children, some were married, and some had no comorbidity or polypharmacy. Our results may not be able to generalize the findings to other cultures and geographic regions in China. Further research on this topic is required to gain deeper insight into these relationships.

## Conclusions

We found that frailty and sleep quality were prevalent and that frailty was positively correlated with PSQI score in elderly patients with OP in China. The higher the frailty score is, the worse the sleep quality. It means the frailer old patients were, the worse their sleep quality, and poor sleep quality may increase the risk of frailty in elderly patients with OP. Therefore, medical staff should pay attention to frailty, sleep quality and its influencing factors, recognizing frailty and poor sleep quality in time in elderly individuals with OP, adopting effective intervention measures, and improving their health and quality of life.

## Data Availability

The datasets generated and analyzed during the current study are not publicly available due to protecting the participants’ personal information but are available from the corresponding author on reasonable request.

## Abbreviations

OP: Osteoporosis
ADLs: Activities of daily living status
BMD: Bone mineral density
BMI: Body mass index
DXA: Dual-energy X-ray absorptiometry
PSQI: Pittsburgh Sleep Quality Index

## References

[1] Borgström F, Karlsson L, Ortsäter G, Norton N, Halbout P, Cooper C (2020). Fragility fracture in Europe:burden, management and opportunities. Arch Osteoporos.

[2] Johnston CB, Dagar M (2020). Osteoporosis in older adults. Med Clin North Am.

[3] He LY, Sun Y, Yao WJ, Pan KQ (2016). The prevalence rate of osteoporosis in the elderly in China between 2010 and 2016, a meta-analysis of single rate. Chin J Osteoporos..

[4] Si L, Winzenberg TM, Jiang Q, Chen M, Palmer AJ (2015). Projection of osteoporosis-related fractures and costs in China: 2010–2050. Osteoporos Int.

[5] Bliuc D, Nguyen ND, Nguyen TV, Eisman JA, Center JR (2013). Compound risk of high mortality following osteoporotic fracture and refracture in elderly women and men. J Bone Miner Res.

[6] Collard RM, Boter H, Schoevers RA, Oude Voshaar RC (2012). Prevalence of frailty incommunity-dwelling older persons: a systematic review. J Am Geriatr Soc.

[7] Ma L, Tang Z, Zhang L, Sun F, Li Y, Chan P (2018). Prevalence of frailty and associated factors in the community-dwelling population of China. J Am Geriatr Soc.

[8] Clegg A, Young J, Iliffe S, Rikkert MO, Rockwood K (2013). Frailty in elderly people. Lancet.

[9] Luo J, Zhu G, Zhao Q, Guo Q, Meng H, Hong Z (2013). Prevalence and risk factors of poor sleep quality among Chinese elderly in an urban community: results from the Shanghai aging study. PLoS One.

[10] Li J, Yao YS, Dong Q, Dong YH, Liu JJ, Yang LS (2013). Characterization and factors associated with sleep quality among rural elderly in China. Arch Gerontol Geriatr.

[11] Van de Straat V, Bracke P (2015). How well does Europe sleep? a cross-national study of sleep problems in European older adults. Int J Public Health.

[12] Lai YJ, Lin CL, Lin MC, Lee ST, Sung FC, Chang YJ (2013). Population-based cohort study on the increase in the risk for type 2 diabetes mellitus development from nonapnea sleep disorders. Sleep Med.

[13] Piovezan RD, Abucham J, Dos Santos RV, Mello MT, Tufik S, Poyares D (2015). The impact of sleep on age-related sarcopenia: Possible connections and clinical implications. Ageing Res Rev.

[14] Van Cauter E, Spiegel K, Tasali E, Leproult R (2008). Metabolic consequences of sleep and sleep loss. Sleep Med.

[15] Everson CA, Crowley WR (2004). Reductions in circulating anabolic hormones induced by sustained sleep deprivation in rats. Am J Physiol Endocrinol Metab.

[16] Yao X, Li H, Leng SX (2011). Inflammation and immune system alterations in frailty. Clin Geriatr Med.

[17] Johar H, Emeny RT, Bidlingmaier M, Reincke M, Thorand B, Peters A (2014). Blunted diurnal cortisol pattern is associated with frailty: a cross-sectional study of 745 participants aged 65 to 90 years. J Clin Endocrinol Metab.

[18] Çavuşoğlu Ç, Deniz O, Tuna Doğrul R, Çöteli S, Öncül A, Kızılarslanoğlu MC (2021). Frailty is associated with poor sleep quality in the oldest old. Turk J Med Sci.

[19] Balomenos V, Ntanasi E, Anastasiou CA, Charisis S, Velonakis G, Karavasilis E (2021). Association between sleep disturbances and frailty: evidence from a population-based study. J Am Med Dir Assoc.

[20] Kumar S, Wong PS, Hasan SS, Kairuz T (2019). The relationship between sleep quality, inappropriate medication use and frailty among older adults in aged care homes in Malaysia. PLoS ONE.

[21] Inoue T, Maeda K, Satake S, Matsui Y, Arai H (2022). Osteosarcopenia, the co-existence of osteoporosis and sarcopenia, is associated with social frailty in older adults. Aging Clin Exp Res.

[22] Xiong M, Liu X, You L, Chen X (2020). Relationship between sleep quality and bone mineral density in urban residents. Zhejiang Da Xue Xue Bao Yi Xue Ban..

[23] Song ZX, Chen CX (2017). Influencing factors of sleep disorders among the elderly with osteoporosis. Chin J Public Health..

[24] Zhang YL, Wang CX, Chen LH, Liu YY, Ye SN, Shen Q (2020). Correlation between frailty and self-efficacy in patients with osteoporosis. Chin J Geriatr Care..

[25] Ma YZ, Wang YP, Liu Q, Li CL, Ma X, Wang YJ (2019). 2018 China guideline for diagnosis and treatment of senile osteoporosis. Chin J Gerontol..

[26] Fried LP, Tangen CM, Walston J, Newman AB, Hirsch C, Gottdiener J (2001). Frailty in older adults: evidence for a phenotype. J Gerontol A Biol Sci Med Sci.

[27] Yan DQ, Huang YX, Chen X, Wang M, Li J, Luo D (2021). Application of the Chinese version of the Pittsburgh sleep quality index in people living with HIV: preliminary reliability and validity. Front Psychiatry.

[28] Zhang C, Zhang H, Zhao M, Li Z, Cook CE, Buysse DJ (2020). Reliability, validity and factor structure of Pittsburgh sleep quality index in community-based centenarians. Front Psychiatry.

[29] Martin P, Keppler AM, Alberton P, Neuerburg C, Drey M, Böcker W (2021). Self-assessment of mobility of people over 65 years of age. Medicina (Kaunas).

[30] Wu C, Smit E, Xue QL, Odden MC (2017). Prevalence and correlates of frailty among community-dwelling Chinese older adults: the China health and retirement longitudinal study. J Gerontol A Biol Sci Med Sci.

[31] Avila-Funes JA, Paniagua-Santos DL, Escobar-Rivera V, Navarrete-Reyes AP, Aguilar-Navarro S, Amieva H (2016). Association between employee benefits and frailty in community-dwelling older adults. Geriatr Gerontol Int.

[32] Wang YJ, Wang Y, Zhan JK, Tang ZY, He JY, Tan P (2015). Sarco-Osteoporosis: prevalence and association with frailty in chinese community-dwelling older adults. Int J Endocrinol.

[33] Tatineny P, Shafi F, Gohar A, Bhat A (2020). Sleep in the elderly. Mo Med.

[34] Lee DR, Santo EC, Lo JC, Ritterman Weintraub ML, Patton M, Gordon NP (2018). Understanding functional and social risk characteristics of frail older adults: a cross-sectional survey study. BMC Fam Pract.

[35] Aspray TJ, Hill TR (2019). Osteoporosis and the ageing skeleton. Subcell Biochem.

[36] Nakai Y, Makizako H, Kiyama R, Tomioka K, Taniguchi Y, Kubozono T (2019). Association between chronic pain and physical frailty in community-dwelling older adults. Int J Environ Res Public Health.

[37] Guerriero F, Reid MC (2020). Linking persistent pain and frailty in older adults. Pain Med.

[38] Rodríguez-Sánchez I, García-Esquinas E, Mesas AE, Martín-Moreno JM, Rodríguez-Mañas L, Rodríguez-Artalejo F (2019). Frequency, intensity and localization of pain as risk factors for frailty in older adults. Age Ageing.

[39] Gutiérrez-Valencia M, Izquierdo M, Cesari M, Casas-Herrero Á, Inzitari M, Martínez-Velilla N (2018). The relationship between frailty and polypharmacy in older people: a systematic review. Br J Clin Pharmacol.

[40] Gosch M, Jeske M, Kammerlander C, Roth T (2012). Osteoporosis and polypharmacy. Z Gerontol Geriatr.

[41] Tornero-Quiñones I, Sáez-Padilla J, Espina Díaz A, Abad Robles MT, Sierra RÁ (2020). Functional ability, frailty and risk of falls in the elderly: relations with autonomy in daily living. Int J Environ Res Public Health.

[42] Sasaki N, Fujiwara S, Yamashita H, Ozono R, Teramen K, Kihara Y (2016). Impact of sleep on osteoporosis: sleep quality is associated with bone stiffness index. Sleep Med.

[43] Mander BA, Winer JR, Walker MP (2017). Sleep and human aging. Neuron.

[44] Gerdhem P, Ringsberg KA, Magnusson H, Obrant KJ, Akesson K (2003). Bone mass cannot be predicted by estimations of frailty in elderly ambulatory women. Gerontology.

[45] Sternberg SA, Levin R, Dkaidek S, Edelman S, Resnick T, Menczel J (2014). Frailtyand osteoporosis in older women–a prospective study. Osteoporos Int.

[46] Swanson CM, Shea SA, Kohrt WM, Wright KP, Cain SW, Munch M (2020). Sleep restriction with circadian disruption negatively alter bone turnover markers in women. J Clin Endocrinol Metab.

